# LeCoder: A large-scale automated coder for coding errors in word-production tasks

**DOI:** 10.3758/s13428-026-02948-8

**Published:** 2026-02-17

**Authors:** Shanhua Hu, Delaney DuVal, Brielle C. Stark, Nazbanou Nozari

**Affiliations:** 1 Department of Psychological and Brain Sciences, Indiana University Bloomington, 1101 E 10th St., Bloomington, IN 47405 USA; 2 Cognitive Science Program, Indiana University Bloomington, Bloomington, IN USA; 3 Department of Speech, Language and Hearing Sciences, Indiana University Bloomington, Bloomington, IN USA

**Keywords:** Speech errors, Automated coding, Semantic similarity, Phonological similarity, Aphasia

## Abstract

Speech errors have been instrumental in advancing our understanding of the architecture of the language production system, the nature of its representations, and its disorders. To be most informative, researchers usually need large amounts of data. Hand-coding such data can be both cumbersome and subjective. This paper presents LeCoder, the first open-source, automated error coder for English word and naming data, which uses a data-driven approach grounded in large-scale corpora to quantify the target–response relationship, allowing it to be flexible, scalable, and generalizable across new datasets. By testing the coder on two datasets from two aphasia labs that have been carefully coded by trained research assistants, we first establish that LeCoder has high accuracy when compared to expert coders, and in certain cases, offers a more logical categorization than human coders. We then show, using robust machine-learning approaches, that LeCoder’s performance generalizes to new participants and items it has never encountered before. Collectively, these findings encourage the use of LeCoder across labs for more objective coding of speech errors, which will, in turn, increase replicability of findings in all subfields of research that use speech error analysis, including neuropsychological research.

## Introduction

Speech errors, referring to the deviations of the spoken responses from the intended meanings (e.g., saying “salt” when one actually means “pepper”), have provided valuable insights into the cognitive processes underlying speech production (e.g., Fromkin, 1973; Garrett, 1975), speech monitoring and control (Freund & Nozari, 2018; Hanley et al., 2016; Nozari et al., 2011), language development (e.g., Pinker, 1991; Stemberger, 1989; Waller et al., 2024), and language disorders (e.g., Howard & Gatehouse, 2006; Hepner & Nozari, 2020; Martin et al., 2006; Meier et al., 2016; Nozari, 2019). In particular, analyses of speech errors in individuals with aphasia (IWA) have significantly advanced our understanding of the architecture of the language production system, informing computational models of how lexical and phonological representations are organized and accessed during the word-level production (e.g., Caramazza, 1997; Dell et al., 1997; Nozari et al., 2010; Rapp & Goldrick, 2000; see Dell et al., 2014, for a review). Moreover, such data have been instrumental in identifying the neural correlates of language production and its deficits (e.g., Basilakos et al., 2015; Dell et al., 2013; Fridriksson et al., 2009; Schwartz et al., 2011, 2012; McCall et al., 2023; see Nozari, 2021 and Shekari & Nozari, 2023, for reviews).

Much of the speech error data in IWA come from picture naming tasks (e.g., Snodgrass & Vanderwart, 1980). To generate reliable error data, many standardized picture naming tasks contain a large (i.e., 100+) number of items. Researchers have traditionally relied on manual classification of such errors, but this process can be time-consuming and prone to subjective judgments, thus reducing consistency and reliability, which, in turn, creates major problems in replicability. This paper proposes a solution to this problem by presenting the first automatic coder of single-word speech errors, which we call LeCoder (linguistic error coder). While LeCoder can be used to code speech errors from any population, we test its performance on IWA, as one of the most challenging populations for this purpose. By testing LeCoder on data from IWA and comparing its performance to that of trained human coders, we demonstrate LeCoder’s ability to capture critical nuances in error data and tackle several problems faced by human coders.

## Speech errors and the problems of manual coding

When presented with a picture to name, IWA may produce a variety of responses. Here, we use a widely accepted categorization proposed for coding data from the Philadelphia Naming Test (PNT; Roach et al., 1996). This test contains 175 normed black-and-white line drawings, and participants are given 30 s to name each item. The response for a trial was defined as the first full attempt at generating a name for the picture. After excluding certain errors such as visual errors (e.g., seeing an elongated object like “asparagus” as a *pen*), naming the wrong object in the picture (e.g., naming *car* when the target is a “garage” with a car in it) and descriptions or tangents (e.g., “I like to eat that”) as well as trial with no responses, the response was identified for categorization into one of the following seven categories in Table 1.

**Table 1:** Examples of the seven categories of speech errors according to the PNT

Target	Response	Category	Definition
cat	cat	Correct (R)	The response word is the same or is a synonym of the target word
cat	dog	Semantic Error (S)	The response word is only semantically related to the target word
cat	mat	Phonological Error (F)	The response word is only phonologically similar to the target word
cat	rat	Mixed Error (M)	The response word is both semantically and phonologically similar to the target word
cat	bread	Unrelated Error (U)	The response is a word that is neither semantically nor phonologically similar to the target word
cat	cak	Phonological Related Nonword (N)	The response is a word that is neither semantically nor phonologically similar to the target word
cat	choko	Phonological Unrelated Nonword (NU)	The response is a nonword that is phonologically similar to the target word

Computational models of language production have shown that these seven categories can be reduced to the malfunction of two critical processing dimensions: *semantic* and *phonological* (Dell et al., 1997). Semantic processing describes the first stage of naming, in which a concept is mapped onto lexical items. Phonological processing describes the second stage of processing, in which the lexical item is mapped onto its segments (i.e., phonemes in spoken and graphemes in written production; Dell, 1986; Levelt et al., 1999). In such a two-stage process, disruption of semantic or phonological processes can create different kinds of errors. Disruption of semantic processing creates semantic errors. Similarly, mixed errors arise from disrupted semantic mapping and are made more probable through the feedback from the segments they share with the target (Dell, 1986). Unrelated words also often arise at this level, but they demonstrate a stronger disruption in semantic mapping than that shown by semantic and mixed errors. The signature of disrupted phonological mapping is nonword errors. Phonological errors can also arise in this stage, but they may also be a product of the wrong lexical item having been selected in the first stage of processing because it has been activated through its shared segments with the target (Nozari et al., 2010; Nozari & Dell, 2013).

The origin of speech errors discussed above shows that coding relies critically on two decisions: (a) are the target and the error semantically similar? and (b) are they phonologically similar? Despite detailed instructions in manuals such as those accompanying the PNT and many hours of training, making reliable and accurate decisions for coding these two dimensions remains difficult, albeit for different reasons. In terms of semantic similarity, the problem is subjectivity. First, semantic similarity has various degrees, and determining a threshold for considering two entities similar is not straightforward. For example, while most people agree that “cat” and “dog” are taxonomically related, it is decidedly harder to judge whether “whale” and “parrot” should be considered related, although they are both animals. Furthermore, there are other kinds of semantic similarity, such as thematic similarity (e.g., “dog” and “bone”; e.g., de Zubicaray et al., 2013; Oppenheim & Nozari, 2024; see Mirman et al., 2017, for a review). Deciding the threshold can be even trickier here (e.g., should “coffee” and “brownie” be considered thematically similar or not?). Since it is virtually impossible to define a threshold for all such cases a priori, coders will inevitably make subjective decisions regarding semantic similarity, leading to discrepancies across labs and research groups.

The problem in judging phonological similarity is of a different kind. Coding manuals usually describe explicit rules for such judgments. For example, under the PNT coding guidelines, a target–response pair is considered phonologically similar if it (1) shares the stressed vowel or the initial or final phoneme; or (2) shares two or more phonemes in any position; or (3) shares one or more phonemes in corresponding positions. There are two issues here. First, remembering these rules and applying them consistently, especially in longer words, is difficult, creating many opportunities for coder errors. Moreover, these rules are, to some extent, arbitrary, meaning that there is no reason to believe that they capture the cognitive processes underlying phonological mapping better than other made-up rules. To these problems, one must add the large number of hours it takes to code speech error data, as a rigorous coding process includes transcription and coding by at least two trained coders, followed by a reconciliation process for each participant.

Taken together, the demanding, subjective, and error-prone nature of speech error coding by human data calls for an automation of the process. The challenge, however, is to ensure that such automation does not sacrifice quality. We next describe our general approach to building and testing an automated coder that can be applied to different datasets and individuals with high accuracy.

## Current project

The goal of the current project was to build a publicly available coder, LeCoder, to automatically code target–response relations at the single-word level for English data. One approach to building such a coder is to implement conventional coding rules (e.g., the PNT rules) for determining phonological similarity into the coder. We have deliberately avoided this approach because (a) it ties the coder to a specific set of rules, and (b) as described in the previous section, such rules may not validly represent the cognitive processes they target. Instead, we adopted a data-driven approach grounded in the large-scale corpus and empirical data to quantify the target–response relationship, allowing LeCoder to be flexible, scalable, and generalizable across new datasets. This approach has four steps: (1) computing semantic and phonological similarity using large corpus-based resources, (2) optimizing thresholds with empirical data, (3) coding the target–response pair based on the obtained thresholds, and (4) assessing LeCoder’s overall performance through cross-validation techniques.

To compute semantic similarity, we used Word2Vec (Mikolov, Chen, et al., 2013; Mikolov, Sutskever, et al., 2013), a neural network-based distributional semantic model that represents each word as a unique high-dimensional vector based on the contexts in which the word appears. The fundamental assumption behind Word2Vec is that words occurring in similar contexts tend to have similar meanings. As a result, this approach can not only detect explicit categorical (i.e., taxonomic) relationships, but also effectively capture implicit thematic relationships between words. For example, the word pair “balloon” and “cake”, although not belonging to the same category, are both strongly associated with the schema of a birthday party. Since they frequently co-occur in similar contexts within the corpus, Word2Vec assigns them relatively similar vector representations, resulting in a high semantic similarity score. Thus, by combining Word2Vec with cosine similarity, LeCoder can objectively assess semantic relationships—both categorical and thematic—that may be overlooked or inconsistently judged by traditional manual categorizing methods.

To quantify phonological similarity, we used a new measure derived from normalized Levenshtein distance (Levenshtein, 1966) between the IPA transcriptions of target and response. Compared to rule-based coding guidelines that categorize responses into binary similar/unrelated groups based on fixed criteria, this new metric provides a continuous measure of phonological similarity by accounting for both shared phonemes and their structural alignment, resulting in a graded and more perceptually relevant assessment. The Levenshtein distance is a widely used metric for quantifying the dissimilarity between two given strings (e.g., Heeringa, 2004; Schepens et al., 2012; Serva & Petroni, 2008). It is defined as the minimum number of single-character edits—including insertions, deletions, and substitutions—that are required to transform one string to the other. For example, transforming the word “cat” to “cap” requires only one edit; thus, the Levenshtein distance between these two words is 1. As the two words become more dissimilar, more transformations are needed, and raw Levenshtein distance increases. An advantage of applying Levenshtein distance for phonological similarity is its sensitivity to the relative position of phonemes, which further implicitly captures the syllabic structure. For instance, when a phoneme appears in the onset of one word and in the coda of another, two edits (a deletion and an insertion) are required, whereas alignment in the same syllabic position requires only a single substitution. This property makes the metric more perceptually and cognitively grounded, as it reflects the sensitivity of syllabic positions in linguistic processes, for example, when segments migrate between two words (e.g., “bad man” → “mad ban”), they tend to preserve their syllabic positions in both spoken (e.g., Warker & Dell, 2006) and other modalities of language production (e.g., Atilgan & Nozari, 2025). Thus, the Levenshtein-based approach provides a fine-grained and more cognitively informed evaluation of phonological similarity.

Semantic and phonological thresholds were uncovered using a training procedure. A large number of possible semantic thresholds were generated, and the best threshold was selected by comparing LeCoder’s binarization of data into semantically similar/unrelated to that of human coders in two separate datasets. The same procedure was carried out for selecting the phonological threshold by binarizing the data into phonologically related/unrelated. Next, LeCoder classified errors into the seven standard categories described earlier. These codes were further compared to human codes to compute the accuracy. Finally, its overall performance was assessed using three cross-validation tests: an overall tenfold cross-validation scheme, followed by two leave-one-out tests investigating generalization to new subjects and items.

## Methods

LeCoder is available for public use at https://github.com/NoLab-IU/LeCoder

## Computing semantic and phonological similarity scores

LeCoder’s input included the target word, the response word, and the International Phonetic Alphabet (IPA) transcription of both. The actual words were used for computing semantic similarity, whereas IPA codes were used for computing phonological similarity scores. For each target–response pair, LeCoder first lemmatized them, then computed a semantic and a phonological similarity score ranging from 0 to 1. To quantify semantic similarity, LeCoder used a Word2Vec model that represents each word as a 300-dimensional vector, obtained from a 100-billion-word corpus (Mikolov, Chen, et al., 2013; Mikolov, Sutskever, et al., 2013). For each target–response pair, semantic similarity is quantified by computing the cosine similarity between their corresponding vector representations. Cosine similarity measures the angle between two vectors, with higher values (closer to 1) indicating greater semantic similarity. In cases where the response word is not presented in the Word2Vec vocabulary, the response is viewed as a nonword response. These cases are automatically excluded from semantic evaluation and are instead routed directly to the phonological similarity computations.

To compute the phonological similarity between the target–response word pair, LeCoder implemented a new measure based on the normalized Levenshtein distance between the words’ pronunciation, transcribed in IPAs described earlier, the Levenshtein distance computes the minimum number of single-character edits required to transform one string to another. The less similar the two strings, the larger the Levenshtein score. For example, transforming the word *water* into *coffee* requires at least five edits, rendering a Levenshtein distance of 5 (Table 2).

**Table 2:** Example of Levenshtein distance computation for “water” → “coffee”

Step	Type	Edition	Current result
1	Substitution	W → C	Cater
2	Substitution	a → o	Coter
3	Substitution	t → f	Cofer
4	Insertion	+f	Coffer
5	Substitution	r → e	Coffee

From the definition, it is evident that the range of the raw Levenshtein distance is larger for longer strings, which can introduce a length-related bias in the computation of phonological similarity. To address this issue, the raw Levenshtein distance was further normalized to account for the impact of the length (i.e., the number of phonemes). The normalized measure was calculated as the ratio of the Levenshtein distance (i.e., the number of edits needed) to the length

of the longer IPA transcription among the two words, yielding a percentage of dissimilarity. The phonological similarity (PhonSim) was then computed as the complement of this ratio (Eq. 1), resulting in a value between 0 and 1, where higher values indicate greater similarity.

1 $$\mathrm{PhonSim}\left(Target,Response\right)=\frac{{\mathrm{L}}_{\mathrm{d}}\left({\mathrm{IPA}}_{\mathrm{T}},\text{ IP}{\mathrm{A}}_{\mathrm{R}}\right)}{\mathrm{max}\left(\mathrm{len}\left({\mathrm{IPA}}_{\mathrm{T}}\right),\mathrm{len}\left({\mathrm{IPA}}_{\mathrm{R}}\right)\right)}$$

where ${L}_{d}$ is the Levenshtein distance, and $IP{A}_{T}$, $IP{A}_{R}$ are the IPA transcriptions for the target and response, respectively.

For example, given the words “cat” and “kitten”, the corresponding IPA transcriptions are/kæt/and/kɪ́tən/, respectively. To transform/kæt/into/kɪ́tən/, at least three single-character edits are required: (1) substitute æ with ɪ́, (2) insert ə at the end of the string, and (3) insert n at the end of the string. The Levenshtein distance between the IPA transcriptions of these two words is 3. Among the two, kitten has the longer IPA transcription, consisting of five phonemes. Thus, the phonological similarity between cat and kitten is calculated as 1 – (3/5) = 0.40. Similarly, given the words cat and dog, the corresponding IPA transcriptions are/kæt/and/dɔɡ/. The Levenshtein distance between these transcriptions is 3, and the maximum length of the two IPA strings is also 3. Thus, the phonological similarity between cat and dog is calculated as 1 – (3/3) = 0.00, indicating that the word pair is not phonologically similar. In short, our approach yielded a semantic and a phonological similarity score for each target–response pair, ranging from 0 to 1, with higher scores representing greater similarity.

## Special cases in coding similarity

While the coding process was largely automated based on web-based resources, there were a few cases that required special attention. These included onomatopoeia, synonyms, hypernyms and hyponyms, compound words, capitonym, and diphthongs. Each case is discussed below.

### Onomatopoeia

In some trials, the participants responded to the given picture using onomatopoeia (i.e., words that imitate sounds, such as “purr” or “nigh”). These responses typically lacked concrete lexical meaning and did not reflect the participants’ ability to retrieve the appropriate lexical item for the target picture. Therefore, they should be classified as NR. To systematically identify onomatopoeic responses, LeCoder implemented a predefined list of commonly occurring onomatopoeia (VOICE, 2021). For each target–response pair, the response word was first compared with the list before proceeding to the semantic similarity computations. If a match was found, then the trial was automatically coded as NR, and the following semantic similarity computation steps were skipped.

### Synonyms

Although many standardized picture naming tasks used pictures with high name agreement, synonyms were not uncommon responses in such tasks. For example, the participant may respond to the target “television” with “TV”*.* In such cases, since the response reflected an accurate understanding and successful naming of the given picture, the trial should be coded as R. To identify synonym responses, LeCoder used WordNet (Fellbaum, 1998) to extract a list of synonyms for each target word. Before proceeding to semantic similarity computations, the response word was compared to the synonym list. If a match was found, then the trial was automatically coded as R, and the following semantic similarity computation steps were skipped.

### Hypernyms and hyponyms

The desired response in naming tasks is usually the basic-level category (e.g., “dog”), which represents an optimal level of abstraction preferred by humans in learning, perception, and memory (Rosch et al., 1976). However, in some trials, participants may respond to the picture with words from different levels of category hierarchy, either more specific (hyponyms, e.g., “Doberman”) or more general (hypernyms, e.g., “animal”) than the basic-level category target words. Rather than broadly labeling these cases as semantic errors, LeCoder provided a more fine-grained categorization by explicitly tagging them as *hypernym* or *hyponym* responses, respectively.

To identify these hierarchical relationships, LeCoder used WordNet to assess potential category-level links between the target and the response. Since such relationships only applied to word pairs that shared the same part of speech (PoS)—and all target words in the task were nouns—this step was performed only when the response is also predominantly used as a noun. Responses that could not function as nouns, or were not primarily nouns, bypassed this step and proceeded directly to the semantic similarity computation. Specifically, LeCoder retrieved the lowest common hypernym (LCH) shared by the target–response pair. If the LCH was equal to the target word, the response was categorized as a hyponym; if the LCH was equal to the response word, the response was categorized as a hypernym (see Table 3). Otherwise, if the LCH did not match either the target word or the response word, it indicated that no direct hierarchical relationship existed between them. In such cases, the word pair proceeded to the next step for semantic similarity computation.

**Table 3:** Examples of hypernym and hyponym classification

Target	Response	Lowest Common Hypernym (LCH)	Tag
dog	Doberman	dog	Hyponym
dog	animal	animal	Hypernym
dog	flower	organism	N/A, proceed to the next step

### Compound words

Compound words are composed of two or more lexical items, such as “cheerleader”, “firefighter”, and “cheesecake”. In the Word2Vec vocabulary, compound words may appear in different forms—for example, as a single word (“cheesecake”) or as multiple words joined with an underscore (“cheese_cake”). This inconsistency can lead to errors for automated coding. To address this, LeCoder attempts to decompose each response word into possible subwords through the WordNinja package (https://github.com/keredson/wordninja). If a valid underscore-joined form exists in the corpus-based vocabulary, LeCoder computes semantic similarity for both the original single-word form and the underscore-joined form. The higher of the two values is selected and used for further classification. Otherwise, if the response cannot be split, the word is treated as a single word and proceeds directly to the semantic similarity computation.

### Capitonym

Capitonyms refer to words whose meanings change when the first letter is capitalized. For example, “father” (all lowercase) typically refers to a male parent, whereas “Father” (first letter capitalized) may refer to a religious figure. Since LeCoder lemmatized all input words to lowercase by default, it may overlook instances where capitalization alters meaning, and thus potentially affect both the computed semantic similarity and the accuracy of the response classification. For example, the semantic similarity score between “church” and “father” was 0.248, whereas the same score between “church” and “Father” was 0.435. To address this issue, LeCoder calculated the semantic similarity between the target word in both forms. The higher of the two similarity scores was then used for threshold comparison during the tagging process.

### Diphthongs

On the phonological side, a primary challenge for LeCoder was correctly handling diphthongs. Diphthongs refer to combinations of two vowel sounds that function as a single phoneme in the IPA transcription. For example, in the word “light” (/laɪt/), the sound/aɪ/is a diphthong and should be treated as one phoneme. Thus, the correct phoneme count for this word is three:/l/,/aɪ/, and/t/. Incorrectly counting diphthongs as two separate phonemes can impact the IPA length and lead to errors in calculating phonological similarity between words. In English, there are eight primary diphthongs:/aɪ/,/aʊ/,/eɪ/,/oʊ/,/ɔɪ/,/ɪə/,/eə/, and/ʊə/. To avoid double-counting, LeCoder substituted all eight diphthongs with a unique single digit before proceeding to the phonological similarity computation. For example, the IPA transcription of the word “light” (/laɪt/) was transformed into the string/l1t/and then proceeded to the next step.

The diphthong substitution ensured that phonemes were counted accurately and that identical diphthongs were consistently treated as the same unit across words. Extending the example of the word “light”, the substituted string/l1t/had a length of three, which correctly reflected the number of phonemes in the word. When compared to the word “lion” (/laɪən/→/l1ən/), the minimum edits required were two: (1) replace/t/with/ə/, and (2) delete/n/. Thus, the Levenshtein distance was 2, and the normalized phonological similarity was calculated as 1 – (2/4) = 0.50, which correctly reflected the fact that “light” and “lion” share the phonemes/l/and/aɪ/.

## Training LeCoder for thresholding

Recall that the ultimate goal of LeCoder is to code responses into the seven categories described above, in a reliable and accurate manner. To achieve this, the continuous similarity scores for semantic and phonological dimensions must first be converted into binary decisions (related or unrelated) in each dimension, and then combined to form the seven categories. Table 4 shows these combinations and the ensuing tags. If the response is not found in the Word2Vec vocabulary, derived from a hundred-billion-word corpus, it is labeled as a nonword, and no semantic similarity can be computed. If it is deemed phonologically similar, it is tagged as N, if not, as NU. All lexical items undergo semantic similarity evaluation. If the item is labeled as semantically related, but not phonologically related, it is tagged as S. If the opposite is true, it is tagged as F. If the item is deemed to be both semantically and phonologically related to the target, it is tagged as M, and if it is deemed to be neither semantically, nor phonologically related to the target, it is tagged as U (see Appendix A for a sample trial).

**Table 4:** Examples of different error types for the target word “cat” along with their semantic and phonological similarity scores and final tags

Target	Response	Semantic Similarity Score	Semantically Similarity Classification	Phonological Similarity Score	Phonological Similarity Classification	Tag
cat	dog	0.7609	Y	0.0000	N	Semantic Error (S)
cat	mat	0.2468	N	0.6667	Y	Phonological Error (F)
cat	rat	0.5328	Y	0.6667	Y	Mixed Error (M)
cat	bread	0.1587	N	0.0000	N	Unrelated Error (U)
cat	cak	N/A	N/A	0.6667	Y	Phonologically Related Nonword (N)
cat	choko	N/A	N/A	0.0000	N	Phonologically Unrelated Nonword (NU)

But converting the continuous similarity scores into a binary (related/unrelated) decision requires setting a *threshold*. A semantic (s) threshold is a cutoff point on the semantic similarity spectrum below which a target–response pair is deemed to be semantically unrelated. Similarly, a phonological (p) threshold is a cutoff point on the phonological similarity spectrum below which a target–response pair is considered to be phonologically unrelated. To uncover the s and p thresholds that maximize LeCoder’s performance, we used a training procedure. This procedure entails a grid search of a large number of s and p thresholds and comparing LeCoder’s classification of semantic and phonological relatedness using each threshold value to the judgments of trained human coders. We employed two datasets coded by trained human coders from two separate aphasia labs. These two datasets use different IWA and different picture sets, providing both subject-level and item-level variability for assessing LeCoder’s performance and its ability to generalize beyond its training set. We first describe the characteristics of each dataset and then explain the training process.

### Empirical datasets

#### Dataset 1 (D1)

D1 had been collected at the University of South Carolina from 38 individuals with chronic aphasia (27 males, mean = 61 years old, SD = 11 years, mostly with Broca’s profile). Participants were administered the Western Aphasia Battery – Revised (WAB; Kertesz, 2006), with the average Aphasia Quotient being moderate-severe: range = 20.1–91.8, mean = 48.49. WAB Fluency on describing a picture and answering interview questions was (maximum score = 10): range = 1–9, mean = 3.08. Auditory comprehension, measured for words, sentences, and sequential commands, was relatively preserved in the sample (maximum score = 10): range = 4.15–9.75, mean = 7.45. Repetition for words and sentences was impaired (maximum score = 10): range = 0.1–8.4, mean = 3.86; as was object naming and word finding (maximum score = 10): range = 0.1–9.3, mean = 4.36. Semantic knowledge was largely preserved, as indicated by a test of noun relationships (Pyramids and Palm Trees Test; Howard & Patterson, 1992, maximum score = 52): range = 30–52, mean = 44.18; and verb relationships (Kissing and Dancing Test; Bak & Hodges, 2003, maximum score = 52): range = 30–41, mean = 44.95. Nonverbal reasoning, measured by the Weschler Adult Intelligence Test – IV Matrix Reasoning (Weschler, 2008) was largely preserved: range = 4–21, mean = 10.34.

Each patient completed up to 12 sessions of the standardized 175-item PNT, with a 30-s response deadline. The target word list had an average length of 5.39 letters (SD = 1.97) and an average of 4.64 phonemes (SD = 1.81). Based on the SUBTLEXus database (Brysbaert & New, 2009), the average base 10 log word frequency of the target words was 2.95 (SD = 0.62). The resulting dataset contained a total of 22,649 target–response pairs from 339 sessions. Each naming task response had been independently transcribed into words by two trained research assistants and subsequently reconciled to produce the final transcript. The first response in each trial had been identified and coded by two independent coders in one of the seven categories, according to the PNT tagging guidelines, as described earlier.

#### **Dataset 2 (D2)**

D2 was collected at the senior author’s former lab at Johns Hopkins University from 12 individuals with chronic aphasia (9 males, mean = 59 years old, SD = 11 years, mostly of Broca’s type). All patients completed an in-house battery, with the WAB fluency test, semantic and lexical comprehension, auditory word repetition using Philadelphia Repetition Test (PRT), and naming using PNT (Roach et al., 1996). The sample characteristics were as follows: WAB fluency (maximum score = 10): range = 5–10, mean = 6.33; semantic comprehension accuracy (conceptual picture matching): range = 93–100%, mean = 97%; lexical comprehension accuracy (auditory word-picture matching with semantic and phonological foils): range = 80–100%, mean = 90%; picture naming accuracy: range = 39–95%, mean = 66%; auditory word repetition accuracy: range = 50–99%, mean = 87%. In short, this sample comprises IWA with good semantic and lexical comprehension and picture naming abilities ranging from mild to moderate severity (individuals with anomic, Broca’s, and conduction aphasia).

Patients completed two sessions of the naming task using a 444-item battery. This dataset used colored pictures from publicly available sources, and participants had 20 s to respond to each picture (see Nozari, 2019, for more details). The resulting dataset contains a total of 10,362 entries. The 444-item battery used in collecting D2 contained 94 words that overlap with those in the 175-item PNT, while the remaining 350 words were not included in the PNT. The target word list had an average length of 6.03 letters (SD = 2.07), an average of 5.36 phonemes (SD = 1.94), and an average base 10 log word frequency of 2.44 (SD = 0.71). Excluding the overlapping words, the unique target word list has an average length of 6.28 letters (SD = 2.07), an average of 5.59 phonemes (SD = 1.94), and an average base 10 log word frequency of 2.30 (SD = 0.69). Similar to D1, each response in D2 was transcribed, coded according to the PNT rules, and reconciled by two trained research assistants. A *t* test comparing word frequency across the two datasets revealed significantly higher frequency in D1 compared to D2 (*t* = 8.58, *p* <.001, Cohen’s *D* = 0.74). Similarly, words in D2 contained significantly more phonemes than D1 (*t* = – 4.34, *p* <.001, Cohen’s *D* = 0.38). These differences highlight the different characteristics of the two datasets and are important for testing generalization (Table 5).

**Table 5:** Description of datasets. WAB = Western Aphasia Battery

	Dataset 1 (D1)	Dataset 2 (D2)
*N*	38 (Male = 27)	12 (Male = 9)
Age	M = 61, SD = 11	M = 59, SD = 11
WAB fluency	M = 3.08, range = 1–9	M = 6.33, range = 5–10
Number of sessions	Up to 12	2
Number of items	175	444
Log word frequency	M = 2.95, SD = 0.62	M = 2.44, SD = 0.71
Phonemes	M = 4.64, SD = 1.81	M = 5.36, SD = 1.94

In order to use the empirical datasets for finding the optimal semantic and phonological thresholds, we reduced the seven response categories to two dimensions, semantic and phonological similarity, and binary coding of target–response pairs for each. To this end, we first excluded NR code. Next, two lists were generated, one for evaluating semantic similarity and one for phonological similarity. For the semantic list, we retained all lexical responses. Trials tagged as N or NU, which involve nonword responses, were excluded from the semantic similarity. Of the retained tags, S and M were labeled as semantically similar, whereas F and U were labeled as semantically unrelated. For the phonological list, tags F, M, N were labeled as phonologically similar, while S, U, NU were labeled as phonologically unrelated. These two lists were used as standards in the next steps for threshold optimization and performance evaluation.

### Training for thresholding

Training was used to determine the optimal s and p thresholds. To identify the optimal semantic threshold, we performed a grid search over 1001 values between 0 and 1, incremented by 0.001. For each candidate threshold, a target–response pair was classified as semantically related if its semantic similarity value was greater than or equal to the threshold and classified as unrelated otherwise. These LeCoder-generated predictions were then compared to a binary standard list generated from human codes discussed in the above section. The threshold that produced the highest agreement between LeCoder’s predictions and manual tags was selected as the optimal semantic threshold for the dataset. The same process was repeated for phonological thresholding.

## Testing LeCoder’s performance

To evaluate the generalizability of LeCoder on new data, we applied the technique of cross-validation. The basic idea behind cross-validation is to systematically divide the dataset into multiple subsets, using most for training and holding out one subset for testing. This process was repeated until each subset had served as the test set. Thus, cross-validation provided a more reliable estimate of LeCoder’s ability to generalize beyond the specific data on which it was trained. Three sets of cross-validation were performed: (1) a standard tenfold cross-validation to assess LeCoder’s overall performance, (2) a subject-level leave-one-out (LOO) cross-validation to assess the generalization of LeCoder’s performance to subjects it has not encountered before, and (3) an item-level LOO cross validation, to assess the generalization of LeCoder’s performance to items it has not encountered before. Each cross-validation was performed both within D1 and D2 and across the two datasets. Since item-level LOO cross-validation requires a large number of repetitions per item, only the 175 items on PNT were used for this test.

**Fig. 1:**
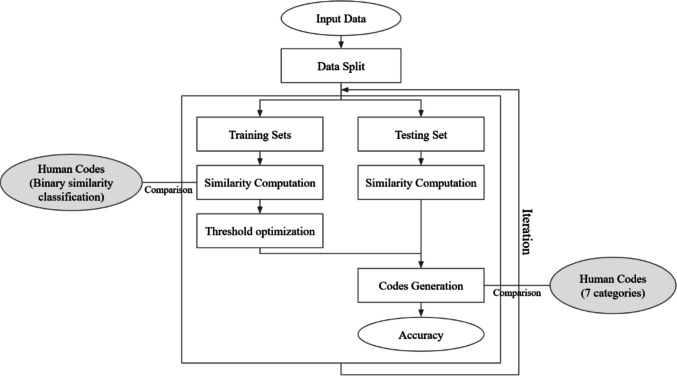
General process for cross-validation

For the general tenfold cross-validation, the dataset was randomly partitioned into ten folds of approximately equal size (Fig. 1, *N* = 10), allowing a maximum size difference of one entry between folds. In each iteration, one fold was used as the testing set, while the remaining nine folds were used for training. During the training phase, semantic and phonological thresholds were computed as described in an earlier section. These thresholds were then applied to the testing fold, and the accuracy was evaluated based on the matches between the predicted final tags and the manual tags in the testing fold. This process was repeated ten times, with each fold serving once as the testing set, ensuring that all data points are evaluated.

For subject-level LOO, the dataset was partitioned based on individual participants (Fig. 1, *N* = 38 for D1, *N* = 12 for D2), with all data from each participant grouped together. In each iteration, the data from one participant was used as the testing set, while the remaining participants’ data were used for training. The semantic and phonological thresholds were derived using the same training procedure described previously and then applied to code the testing participant’s responses. Accuracy was evaluated by comparing LeCoder-generated tags with the manual annotations. This process was repeated until each participant had been used as the testing set exactly once.

For the item-level LOO, the PNT data was partitioned by target word (Fig. 1, *N* = 175), grouping all responses to the same word together. In each iteration, the semantic and phonological thresholds were derived from the remaining data and applied to one group of responses corresponding to the same target word. Accuracy was counted by comparing LeCoder’s predictions with the manual annotations. This process was repeated until each target word had served as the testing set exactly once.

## Results

### Thresholding

**Fig. 2:**
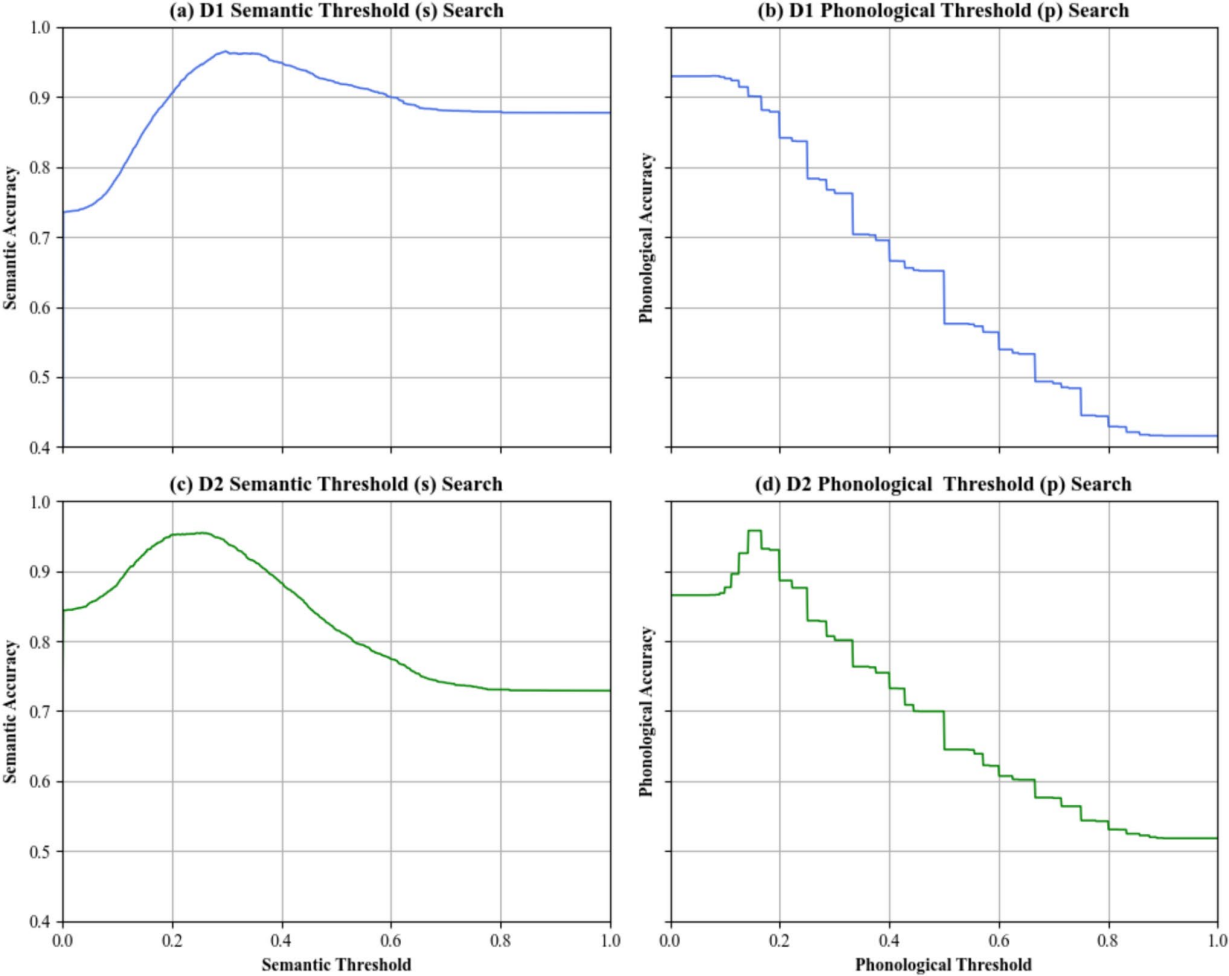
Results of the Threshold Search. LeCoder’s accuracy levels are shown for different levels of s threshold in D1 (**a**) and D2 (**c**), as well as for different values of *p* threshold in D1 (**b**) and D2 (**d**)

Following the training procedure described in the previous section, the optimal semantic threshold for D1 was 0.298, resulting in a 96.87% accuracy for semantic similarity classification (Fig. 2a). The optimal phonological threshold for D1 was 0.084, resulting in a 93.60% accuracy for phonological similarity classification (Fig. 2b). Using this set of optimal thresholds, LeCoder achieved an overall annotation accuracy of 90.00% on D1, as measured by the agreement between LeCoder-generated tags and the manually assigned tags. Performance was strikingly consistent when LeCoder was trained on D2: the optimal semantic threshold for D2 was 0.257, resulting in a 94.94% accuracy for semantic similarity classification (Fig. 2c). The optimal phonological threshold for D2 was 0.143, resulting in a 97.10% accuracy for phonological similarity classification (Fig. 2d). Applying this set of optimal thresholds, LeCoder achieved an overall annotation accuracy of 89.82% on D2.

Two findings are noteworthy. First, s thresholds are generally more stable than p thresholds. For both datasets, accuracy remained above 70% across a wide range of s thresholds, whereas p thresholds greater than 0.4 quickly pushed LeCoder’s performance to chance level. The reason, most likely, lies in the forms of distribution. While the semantic similarity distribution is more evenly spread on the range, the phonological similarity distribution is heavily right-skewed because most word pairs have near-zero phonological similarity (see Appendix B). The more balanced semantic similarity distribution ensures that changes in the threshold have a gradual impact on coding accuracy. In contrast, the concentrated nature of phonological similarity distribution means that increasing the threshold beyond 0.4 reclassifies many similar pairs as unrelated, resulting in a sharp drop in accuracy. Thus, the differing distributional characteristics of the semantic and phonological spaces directly influence their respective threshold sensitivities.

The second finding worth noting is that the s threshold was relatively stable across datasets (0.298 for D1 and 0.257 for D2), whereas the p threshold was markedly different (0.084 for D1 and 0.143 for D2). Recall that both lexical frequency (which has a locus on both stages of word production; Kittredge et al., 2008) and length (which is localized to the second stage of word production; e.g., Meyer et al., 2003) were significantly different between D1 and D2 with effect sizes ranging from medium to large. Thus, the stability of s threshold marks its relative resilience against specific characteristics of the dataset. In contrast, p threshold is clearly sensitive to length. Since LeCoder applies the normalized Levenshtein distance to quantify the phonological similarity, longer words tend to yield higher similarity scores for the same number of phoneme mismatches. Consequently, D2 (which contained longer words than D1) required a higher threshold to distinguish phonologically similar pairs.

## Testing LeCoder’s performance

### General performance

**Fig. 3:**
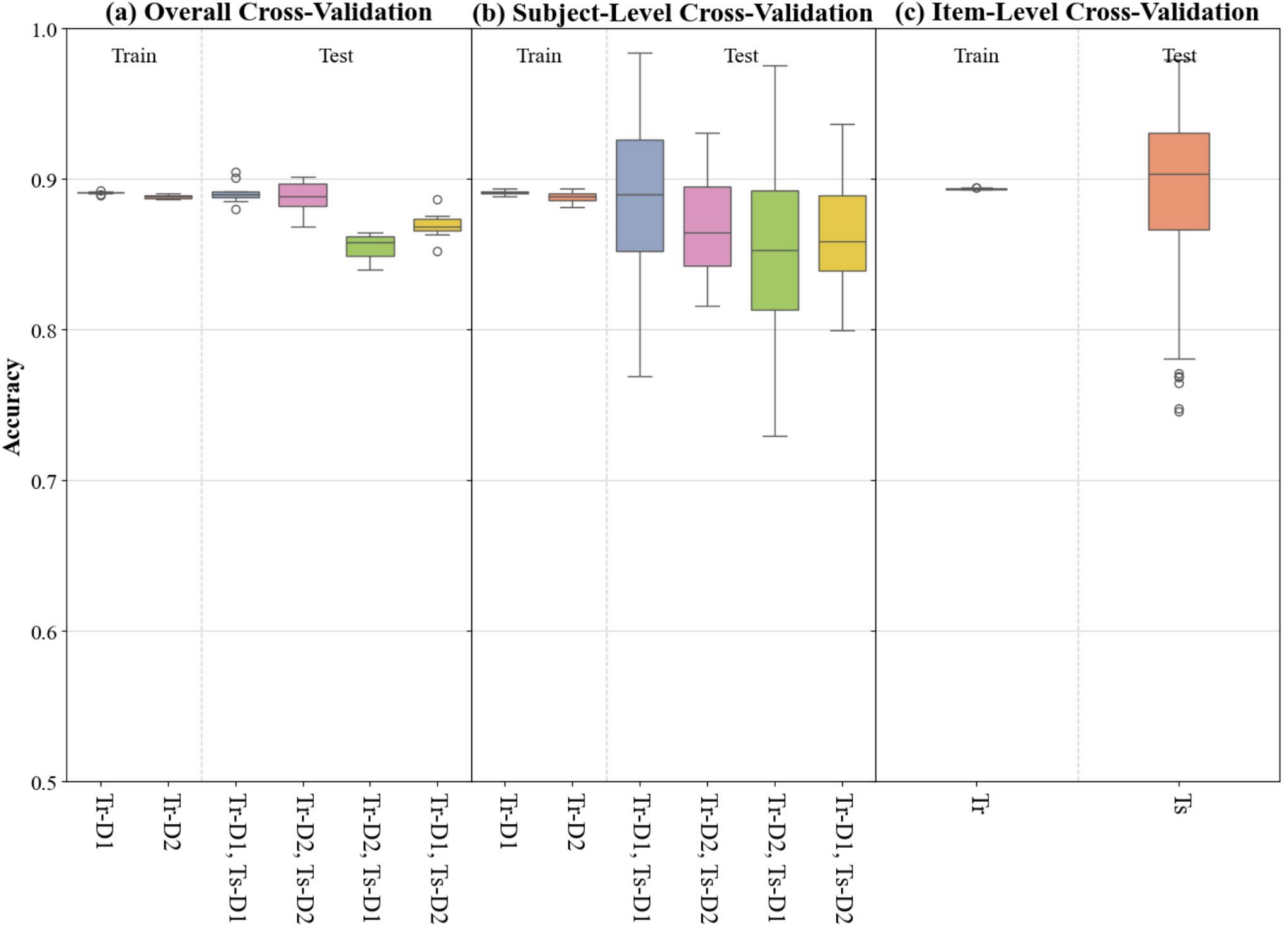
Results of cross-validation tests. Tenfold cross-validation (**a**), subject-level leave-one-out (**b**), and item-level leave-one-out (**c**). Tr = training dataset, Ts = testing dataset. *Whiskers* show the most extreme data points within 1.5 times the interquartile range (IQR) from the box

Figure 3a shows the overall tenfold cross-validation train accuracy (left) and test accuracy (right). The average training accuracy was 89.09% (range = 88.00–89.19%, SD = 0.06%) for D1 and 88.84% (range = 88.59–89.14%, SD = 0.16%) for D2. The average testing accuracy within dataset was 89.09% (range = 88.26–89.93%, SD = 0.52%) for D1 and 88.83% (range = 86.13–91.06%, SD = 1.47%) for D2, indicating a good generalization to unseen data drawn from the same source. To further evaluate LeCoder’s cross-dataset generalizability, the thresholds obtained by training LeCoder on one dataset were applied to the other dataset. The resulting average testing accuracy was 85.51% (range = 84.81–86.89%, SD = 0.61%) for D1 and 86.93% (range = 85.43–89.25%, SD = 1.11%) for D2. These findings suggest that LeCoder’s performance was stable across datasets and showed a high level of agreement with manual annotations.

### Subject-level generalization

Figure 3b shows the subject-level LOO train accuracy (left) and test accuracy (right). The average training accuracy was 89.09% (range = 88.83–89.37%, SD = 0.11%) for D1 and 88.80% (range = 88.12–89.41%, SD = 0.37%) for D2. The average testing accuracy was 88.61% (range = 76.92–98.39%, SD = 4.61%) for D1 and 86.86% (range = 81.58–93.09%, SD = 3.80%) for D2, indicating strong generalization to data from unseen participants within the same experimental setting. Cross-dataset testing also yielded relatively high performance, with an average testing accuracy of 85.03% (range = 72.94–97.58%, SD = 5.52%) for D1 and 86.35% (range = 79.93–93.68%, SD = 4.12%) for D2. Compared to the general tenfold cross-validation, subject-level LOO cross-validation exhibited greater variability in accuracy. This is expected because of the diverse profiles of the IWA included in each dataset. Despite this, the average accuracy remained high, suggesting that LeCoder is robust across diverse participant profiles.

### Item-Level generalization

Figure 3c shows the item-level LOO train accuracy (left) and test accuracy (right). Recall that this analysis could only be performed on the PNT data, therefore the full set of analyses reported for 10-fold and subject-level LOO is not available for this analysis. The average training and testing accuracy was 89.35% (range = 89.30–89.46%, SD = 0.03%) and 89.34% (range = 74.54–97.94%, SD = 5.16%), respectively. This indicates strong performance of LeCoder on data containing previously unseen target words.

#### **Discrepancies between human and LeCoder codes**

As evident in Fig. 3c, while most target items achieved accuracies above 80.00%, a few outlier items exhibited relatively lower accuracy, around 75.00%. Therefore, we inspected these items more closely to uncover the reason behind the systematic discrepancy between LeCoder and human codes. Table 6 shows these cases.

**Table 6:** Outlier items with divergent codes. *There was no common error for Eskimo, as the problem was that some participants were presented with a different target picture for that trial

Target	Common response		Human code	LeCoder code	Semantic Similarity score
ball	bowl	Phonological Error (F)		Mixed Error (M)	0.3280
pig	big	Phonological Error (F)		Mixed Error (M)	0.3123
bread	bed	Phonological Error (F)		Mixed Error (M)	0.3449
cross	church	Semantic Error (S)		Unrelated Error (U)	0.2547
plant	flower	Mixed Error (M)		Phonological Error (F)	0.2845
Eskimo	NA*	NA		NA	NA

We identified six items that were consistently coded differently by humans and LeCoder. Three cases consist of target-error pairs that LeCoder consider semantically related (as well as phonologically related) but human coders consider to be only phonologically related. These include “ball-*bowl*”, “pig-*big*”, and “bread-*bed*”. LeCoder’s behavior is justifiable for these items. “Ball” is associated with “Super bowl”, and “bowling”. As such, there is a semantic relationship between this target and error. Similarly, “*big*” is an adjective often used for “pig”, marking a thematic association. Since PNT guidelines limit responses to nouns, human coders did not consider this association, but producing responses in semantic categories other than nouns is not at all unusual in IWA. Finally, “*bed*” and “bread” are coded as semantically related, most likely due to the mediating item “breakfast”. “*Bed*” is highly associated with “breakfast” through the common conjoined noun-phrase “bed and breakfast”, and “breakfast” with “bread”.

However, there are also cases where LeCoder’s judgment is incorrect. This is evident in target-error pairs “cross-*church*” and “plant*-flower”,* where LeCoder misses the obvious semantic relationship. In the former case, the issue is that “cross” is a homophone with very different meanings and contextual diversity. Since LeCoder has no access to the visual context, it considers all meanings and contexts of “cross”, including the mathematical symbol for multiplication, the act of traversing a plane (crossing the street), most of which are semantically unrelated to “*church*”. As a result, it assigns a borderline semantic similarity score of 0.255, leading to a classification of semantically unrelated. In the latter case, the limitation comes from WordNet’s taxonomical hierarchy. While the “plant-*flower*” is a hypernym-hyponym relationship, WordNet does not encode this specific link and instead identifies the LCH (lowest common hypernym) of the pair as the overly broad category “whole.” As a result, LeCoder cannot detect the intended hierarchical relationship and fails to classify the response “*flower*” as a hyponym of the target word “plant”. Finally, the sixth case represented a technical error in D2. We realized that in a subset of participants in this dataset, responses to the target “Eskimo” were attempts at producing a different target word, “umbrella”, because those participants had, in fact, seen a picture of an umbrella and not an Eskimo. Naturally, their responses had been coded by the human coders with “umbrella” as the target. As such, LeCoder’s classification of these errors as unrelated to “Eskimo” reflect LeCoder’s correct judgment given the target it assumed for the response.

In short, when LeCoder identified a semantic relationship underlying the target-error pairs and human coders did not, LeCoder’s judgment was justifiable and perhaps better reflective of the representation of semantic similarity than human coder’s judgments. When it failed to identify a semantic relationship that human coders had identified, the problem was, in one case, a homophone with a very high contextual diversity, and in another, an error in WordNet. While these two cases were clear errors, they were rare compared to LeCoder’s high performance and its good generalization to new subjects and items.

## Discussion

This project proposed an automatic coder that could accurately and reliably code word-level errors in English data across a wide range of items and subjects. The results showed that LeCoder’s performance aligns well with that of trained human coders and generalizes well to new participants and items the coder has not been exposed to before. A closer examination of cases where LeCoder’s performance deviated systematically from that of human coders revealed that such cases were rare, and in some cases, LeCoder’s judgment was better justified than human coders’ judgments. These results highlight LeCoder’s capacity to maintain consistent coding accuracy, reinforcing potential utility for broader applications in speech error annotation across datasets. Below, we recapitulate LeCoder’s advantages and end by discussing future steps in addressing LeCoder’s limitations.

## Advantages of LeCoder over human coders

Regarding the judgment of semantic similarity, LeCoder produces annotations that are both more objective and more exhaustive compared to the traditional manual coding process. Specifically, LeCoder produces consistent judgments when presented with identical target–response pairs and is capable of identifying a wide range of semantic relationships, including those involving rare or domain-specific words. Inspection of the human codes revealed that even trained coders from the same lab did not always assign consistent codes to the same target-error pair across participants, causing inconsistent and unreliable coding. Moreover, certain low-frequency responses, e.g., “snake → *asp*” were coded as “unrelated” by human coders, presumably because they did not know that “asp” is a specific kind of snake.

In addition, the PNT coding guidelines restrict the semantic similarity judgments to target–response pairs that share the same part-of-speech tag, namely nouns. As a result, if the participant responds with a verb or adjective, the manual coder is required to tag the trial as semantically unrelated, regardless of the meaning and the underlying relationship of the response. However, it is observed from the dataset that in many cases, such non-noun responses clearly reflect conceptual understanding and subsequent lexical access of related items. The “ball → *bowl*” example, discussed as one of the common deviations of LeCoder’s judgment from human coders, may reflect a case where the semantically related “bowl” was considered to be a verb by the human coder and thus excluded as a viable candidate for assessing semantic similarity. Similarly, “pig → *big*” is an example of ignoring an underlying semantic-lexical association, on top of phonological similarity, which contains important information regarding the state of the production system. In contrast to human coders, LeCoder is not constrained to noun responses, thus enabling the coder to capture more fine-grained nuances in semantic and lexical associations in the speaker’s language production system.

In terms of phonological similarity judgements, LeCoder computes similarity in a way that is too complicated for human coders, and yet yields results that are intuitive and hold irrespective of word length. The problem with PNT rules is that, despite their surface consistency, their disregard for length effects creates problems. For example, consider the rule for coding a pair as similar if they share “two or more phonemes (including stressed vowels but excluding unstressed vowels) in any position”. This rule would consider both “top → *pot*” and “telescope → *hippopotamus*” as phonologically similar. However, the overlap in the former pair is an example of a transposition with a shared vowel that is likely to create real phonological confusion (e.g., Toscano et al., 2013), whereas the latter is much more likely to be simply due to chance. With the normalized Levenshtein distance, the LeCoder calculated phonological similarity between “top/tɑp/→ *pot*/pɑt/” as 1 – (2/3) = 0.33, which exceeds the threshold and thus is coded as phonologically similar. In contrast, “telescope/tɛləskoʊp/→ *hippopotamus*/hɪpəpɑtəməs/” yields a similarity of 1 – (10/11) = 0.09, which falls below the threshold of 0.143 derived from D2 with a larger average phoneme count, resulting in a phonologically unrelated code. The difference between the phonological similarity codes generated by PNT rules and LeCoder demonstrates that, by accounting for word length, LeCoder can more reliably distinguish meaningful phonological similarity from coincidental phoneme overlap, thereby providing codes that better reflect the potential cognitive assessment of phonological similarity.

In addition to taking length into account, LeCoder also considers syllabic position in its computations, which is important in phonological encoding. Psycholinguistic studies have shown that primes with some degree of phonological overlap with targets facilitate target processing (e.g., Radeau et al., 1995), but this facilitation appears to hinge on positional overlap between the shared segments of primes and targets. For example, Gagnon (1994) conducted an auditory priming task and found that a reversed order of phonemes between primes and targets led to inhibition, rather than facilitation, of target production (see also Gagnon & Sawusch, 1989). These findings highlight the importance of computing phonological similarity in a manner that accounts for positional similarity. The normalized Levenshtein scores in LeCoder fulfill this requirement.

## Limitations and future path

Despite the advantages discussed above, the current implementation of LeCoder has several limitations that suggest possible directions for future development. First, the coder does not take the visual information of the actual picture shown to the participants, and thus cannot constrain the target word to a single correct meaning. In the majority of cases, LeCoder handled this well. However, there were occasional cases where the absence of visual constraint led to systematic errors. For example, LeCoder’s computation of semantic similarity between “cross → *church*” consistently fell below the s threshold, leading LeCoder to classify this pair as unrelated. As mentioned earlier, the problem is semantic diversity; when LeCoder considers all possible meanings of a word with very diverse meanings —in this case ranging from a religious symbol to a mathematical operator to a motion verb— target-error cooccurrence can be significantly diluted by considering the irrelevant contexts. Our findings showed that LeCoder is, to a large extent, robust against such semantic diversity, but to completely eliminate occasional misclassifications such as the above example, future work could integrate multimodal models to align visual and linguistic inputs for better semantic judgment. For example, recent models such as CLIP (Contrastive Language-Image Pretraining; Radford et al., 2021) and Flamingo (Alayrac et al., 2022) have demonstrated outstanding performance in aligning images with corresponding language representations, which means they are capable of interpreting visual input and helping narrow down the lexical meaning. Future work can incorporate those models into LeCoder to enable conditioning its semantic similarity judgments on the specific referential meaning associated with the visual stimulus, and thus further provide contextual disambiguation to improve performance on visually grounded naming tasks.

Second, LeCoder uses information from large-scale publicly available sources. On the one hand, this is a great strength, as it makes LeCoder a modern, up-to-date, and scalable automatic coder. On the other hand, similar to any other large-scale tool that taps into open-source data, LeCoder’s output relies on the accuracy of its sources. Our results show that such sources are, for the most part, accurate and reliable. However, occasional mistakes in WordNet will inevitably affect LeCoder’s output. For example, if hyponym/hypernym relationships are misclassified in WordNet (e.g., plant/flower), LeCoder will produce the wrong classification. This problem does not have an immediate solution, but the general structure of LeCoder allows for easy updates and pivots to new sources, as public sources are refined and better sources emerge, further contributing to its versatility and potential for continued large-scale use.

Finally, although the current version has been tested on two datasets with considerable subject and item variability, there is definitely room for improvement through testing LeCoder’s performance on other populations (e.g., children) and items. By making the tool publicly available to all researchers interested in working with linguistic error data, we hope to tap into the real potential of Open Science by receiving feedback about LeCoder’s current shortcomings and addressing them in future updates.

## Conclusion

This study proposed and evaluated LeCoder, the first automated speech error coder that applied a data-driven approach with large-scale corpus-based representations and empirical experimental data to efficiently code the target–response relationship in word production tests. The results demonstrated that the LeCoder’s accuracy is close to the accuracy of trained human coders, validating its reliability as an error coding tool. Moreover, the coder showed strong generalizability when applied to new participants and/or unseen target words, suggesting its stability across diverse datasets. These findings highlight the potential of LeCoder as a public tool for fast and reliable coding of linguistic errors across labs and research groups. In addition to providing a solid platform for improved coding of English linguistic errors, the pipeline proposed in this paper can be easily adapted to many other languages, encouraging the development of scalable tools in those languages as well.

## Figures and Tables

**Fig. 4.**
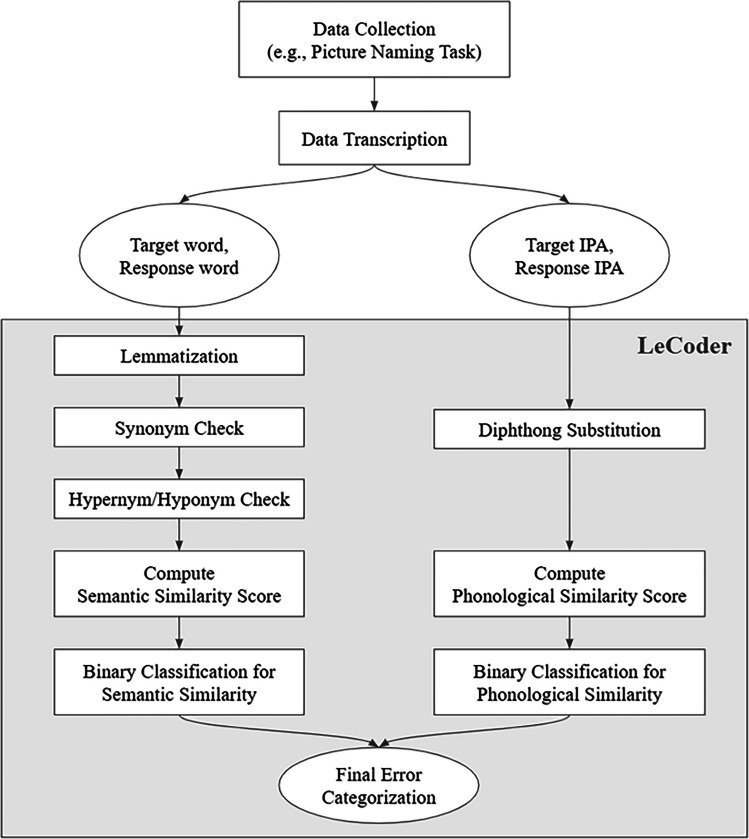
LeCoder’s workflow for error coding. All operations within the gray box are automated

**Fig. 5.**
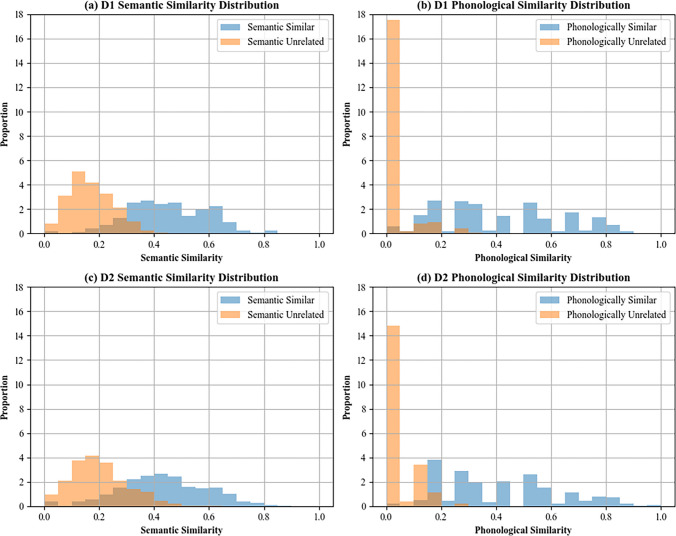
Semantic similarity and phonological similarity distribution

## Data Availability

The datasets analyzed during the current study are available in the GitHub *LeCoder* repository: https://github.com/NoLab-IU/LeCoder/blob/main/LeCoder_Data.csv

## Appendix A

### A sample trial coded by LeCoder

In this section, we will walk through a sample trial step by step to illustrate the full annotation process of this automated speech error coder. Suppose the target word is “dog”, and the participant responds with the word “light”. On the semantic side, the coder first checks whether the response word appears in the predefined onomatopoeia list. Since “light” is not included, the trial is not classified as NR, and the process proceeds to the synonym check. Using WordNet, the synonym list for the target word “dog” includes only one entry: “canine”. As “light” does not match any synonym, the response is not coded as R. Next, the coder checks for a hierarchical relationship. The lowest common hypernym (LCH) of “dog” and “light” is “physical entity”, which does not match either word, indicating no hypernym or hyponym relationship. The process then moves on to compute semantic similarity between the target-response words. Since neither “dog” nor “light” is a compound word that can be split into meaningful components, no further decomposition is applied. The semantic similarity is calculated by comparing the cosine similarity between the vector representations of the word pairs (“dog”, “Light”) and (“dog”, “light”), and the higher value is selected. In this case, the resulting similarity score is 0.248. Compared to the semantic threshold established during the training phase, the two words are considered semantically unrelated.

On the phonological side, the corresponding IPA transcriptions/dɔɡ/and/laɪt/are generated for the target word “dog” and the response “light”. To ensure accurate phoneme counting and alignment, each diphthong is substituted with a unique single-digit placeholder. In this case, the transcription for “dog” (/dɔɡ/) contains no diphthongs and remains unchanged. In contrast, “light” includes the diphthong/aɪ/, which is replaced with a single digit, resulting in the modified transcription/l1t/. The Levenshtein distance is then computed between these two modified strings. To transform/dɔɡ/into/l1t/, three edits are needed: (1) substitute/d/with/l/, (2) substitute/ɔ/with/1/, and (3) substitute/ɡ/with/t/, for a total of three edits. Given that the longer transcription between the two words has a length of 3, the normalized phonological similarity is calculated as 1 – (3/3) = 0.00. According to the phonological threshold established during the training phase, this low similarity indicates that the two words are phonologically unrelated.

Lastly, combining the decision from the semantic side (i.e., semantic unrelated) with the judgment from the phonological side (i.e., phonologically unrelated), the final annotation for the target word “dog” and the response “light” is U as unrelated. Figure 4 presents the full workflow for using LeCoder.

## Appendix B

### Semantic and phonological similarity distributions

The semantic similarity distribution is shown for the target-response pair in D1 (**a**) and D2 (**c**), as well as for the phonological similarity distribution in D1 (**b**) and D2 (**d**) Fig. 5.

## Code availability

The compiled program is available in the GitHub *LeCoder* repository: https://github.com/NoLab-IU/LeCoder
